# Unleashing compassionate care: canine-assisted intervention as a promising harm reduction approach to prisonization in Canada and its relevance to forensic psychiatry

**DOI:** 10.3389/fpsyt.2023.1219096

**Published:** 2023-08-03

**Authors:** Maryellen Gibson, Colleen Anne Dell, Darlene Chalmers, Grace Rath, Mansfield Mela

**Affiliations:** ^1^One Health and Wellness Office, Department of Sociology, University of Saskatchewan, Saskatoon, SK, Canada; ^2^Faculty of Social Work, University of Regina, Regina, SK, Canada; ^3^The Centre for Forensic Behavioural Science and Justice Studies, University of Saskatchewan, Saskatoon, SK, Canada

**Keywords:** prisonization, animal-assisted interventions, therapy dog, canine-assisted interventions, harm reduction, compassionate care, incarceration

## Abstract

In recent years, there has been a global advancement in the offering of canine-assisted interventions (CAI) in prisons. However, these programs have focused primarily on the benefits to the dogs involved and not on the impact on the participants. The authors of this perspective study have been running a CAI program with therapy dogs, called PAWSitive Support, in a Canadian federal prison since 2016. Thoughts from the program facilitators and interviews with prison staff indicate that the program, and specifically the therapy dogs, provides a unique and integrated source of comfort, support, and love for participants. These benefits are consistent with those seen in CAI programs outside of prisons. Unique to the prison setting appears to be an improvement in participant–staff relations. The therapy dogs have helped participants to experience comfort and consequently express their emotions. This seems to contribute to their recognition of support within the prison system and specifically developing trust with staff. Additionally, the dogs have helped to create an experience of the feeling of love within the prison, interpreted as the feeling of being cared for, which is rare for this population. The authors suggest that the integration of a therapy dog intervention in prison could be a novel harm reduction strategy to address issues related to prisonization and associated mental health concerns, including substance use. This consideration can offer unique insight into the field of forensic psychiatry about providing compassionate care to patients.

## 1. Introduction

During a presentation on canine-assisted interventions (CAIs) for the International Forensic Psychiatry Lecture Series in 2022 (1), Dr. Dell (who is an author of this study) was struck by a comment made by the series chair: Forensic psychiatry should provide more compassionate care to its patients. This idea has also been suggested in other studies within the forensic psychiatry field (2, 3) as well as in discussions surrounding prisons (4, 5). During her presentation, Dr. Dell suggested that with recent global advancements in the offering of CAIs, this may be one way for forensic psychiatry to consider the provision of more compassionate care to patients.

There is evidence to suggest that interacting with animals can have a positive impact on human health (6–9). This is supported by research specifically focused on animal-assisted interventions (8, 10–14), which “are goal-oriented and structured interventions that intentionally incorporate animals for the purpose of therapeutic gains and improved health and wellness” (15). Animal-assisted interventions are implemented in a variety of settings, such as retirement facilities, university campuses, and prisons. In prisons, CAIs can include training programs for shelter dogs to be rehomed, intensive training for service dogs, canine boarding and grooming services, and programs involving therapy dogs.

Therapy dogs involved in CAIs are friendly companion animals who, alongside a human handler, have passed a test enabling them to visit with individuals who do not have regular access to a dog and can benefit from their comfort, support, and love (16–19). While therapy dog programs commonly result in comfort, support, and love, these outcomes are not clearly defined (19). For instance, university students have defined love with therapy dogs as reciprocal and gaining positive feelings during visits, while support is understood as the creation of a relaxing environment to help students feel less stressed (20). Therapy dogs have also been found to provide comfort, soothe distress, and create a supportive environment where participants feel safe (21). Moreover, therapy dogs have been perceived to offer unconditional and non-judgmental love toward strangers, something not commonly found in initial human-to-human interactions (21–25).

Program evaluation research has demonstrated that CAIs implemented in correctional facilities can have a positive effect on both the institutional atmosphere and participant behavior (8, 26–30). Such interventions can lead to a calmer prison environment, alleviate tensions, and decrease levels of aggression and infraction rates (31–34). The few systematic reviews of CAIs that exist have found that these types of programs can decrease stress and anxiety experienced by participants, improve their levels of self-esteem, and improve participant–staff relations in the short term (27, 29, 35, 36). CAIs can also improve the ability of participants to develop relationships with other humans, including prison staff, through the creation of a trusting and caring relationship with a dog (29, 37). This development of relationships has been found to translate from the connection with the dog into recognition of personal feelings and emotions leading to better conduct within correctional environments (38).

In the realm of substance use research, companion animals and CAIs have been found to increase clients' experiences of connection and care with another being. These feelings of connection, support, love, and comfort can be seen to influence addiction and mental health outcomes including benefiting positive thinking patterns, improved emotional states, and positive attachments and style of attachment (7, 39–41). In 2017, a national survey of Canadians living in recovery found that 88% of respondents identified animals or pets as a support in their recovery journey from substance use with 44% noting animals or pets as important in sustaining their recovery (42). Research is building to suggest that the human–animal bond, the mutually beneficial relationship and connection between humans and animals, can positively influence attachment patterns, a noted factor in substance use recovery (39, 43).

Historically, prisons in Canada, as in many other places, have been designed with a focus on punishment rather than rehabilitation (44, 45). This trend has continued into the present day, with some experts suggesting that it has intensified over the past decade (46, 47). Many correctional institutions operate within a culture of violence, trauma, mistrust, and survival, which can have harmful psychological effects on individuals in these environments (46, 48). This culture and its associated norms can lead to an internalization process called “prisonization” (8, 27, 46, 49, 50). Studies have found that prisonization can contribute to an increase in feelings of stress, anxiety, hypervigilance, distrust, emotional restraint, emotional withdrawal, diminished self-worth, and an overall decline in perceived mental health status (46, 48). There is a considerable representation of mental health concerns within the prison environment with some members of this population requiring forensic psychiatric support (51).

A CAI that involves therapy dogs has the potential to provide prisoners with a distinctive form of comfort, support, and love. Although there is no standard definition of compassionate care at present in healthcare, there seem to be important elements including an aim to reduce the pain, suffering, and harm experienced by a patient and treating patients with compassion, kindness, respect, and dignity (52–54). These elements align closely with harm reduction principles as well, which aim to promote the wellbeing, rights, and dignity of individuals regardless of their socio-economic status, health condition, or location (55, 56). This study reflects on a CAI called PAWSitive Support that has been offered at Drumheller Institution, a medium security prison, in Alberta, Canada, since 2016. Staff have been involved throughout the program's development, providing a unique lens to consider the program's implications. In this perspective study, interviews by the program facilitators with Drumheller Institution staff offer insight into how the PAWSitive Support participants experienced a sense of comfort, support, and love; each contributed to a more affable environment to live and potentially develop within. Based on the staffs' insight and our team's experiences, it is our opinion that CAIs involving therapy dogs could be an innovative harm reduction strategy for addressing issues related to prisonization and associated mental health concerns, including substance use. It is difficult, if not impossible, for individuals to personally develop in an inhospitable environment. This consideration can offer unique insight into the field of forensic psychiatry about providing compassionate care to their patients.

## 2. The PAWSitive support canine-assisted learning program

PAWSitive Support is a canine-assisted learning program that aims to improve the wellbeing of participants through human–canine interaction. It was first delivered at the request of the Warden of Drumheller Institution in response to an increasing number of illicit drug overdoses. The program was developed by Dr. Colleen Dell from the University of Saskatchewan and Dr. Darlene Chalmers from the University of Regina and is grounded in an evidence-based experiential learning approach (57). The program curriculum includes objective-driven activities that are designed to promote human development skills, personal growth, and overall mental health, including addiction recovery. The program involves hands-on work with the program therapy dogs through structured activities that incorporate basic canine training, information about dogs, and the human–animal bond, as well as unstructured time for play and relaxation.

The program is offered over 4 intensive days and includes a follow-up session 6 months later. The 4-day program was designed around space availability. The follow-up sessions were less structured in nature than the initial program and allowed the program participants to reconnect with the program dogs, facilitators, and each other. After completing the program, the participants were invited to return and act as peer mentors. In this role, the peer mentors guided new program participants through the process, offered their insights or ideas on interacting with the program dogs, and supported the current participants in reflecting on the impact of the program on their experiences within prison. The first group of participants consisted of five individuals with a history of substance use, most of whom were serving life sentences. All participants liked dogs, were assessed as non-violent and non-aggressive at the time of the intervention, and had no history of animal abuse. The dogs involved in the program—Kisbey, a loving mature boxer, Subie, an emotionally attuned boxer, and Anna-Belle, a stubborn but affectionate English bulldog—were selected for their friendly personalities and ability to provide comfort, support, and love to those they interact with. They also worked as therapy dogs in their local community. The dogs were assessed for their health, they had basic obedience skills, and their welfare was paramount throughout the program's offering. Two program facilitators, Dr. Colleen Dell and Dr. Darlene Chalmers, led the program activities.

## 3. Insight and future directions for forensic psychiatry

The two program facilitators conducted eight interviews and a focus group with five prison staff members (including administration, front-line, and programming staff) who were associated with the case files of the first five PAWSitive Support participants. The eight individual interviews were conducted between one staff member and one facilitator, with the focus group having both facilitators present. These took place immediately after the program began and then approximately every 6 months over a 2-year period after the program ended to determine program impacts. The interviews and focus groups were semi-structured to allow for insights from the staff members to be collected beyond the experiences or knowledge of the facilitators and varied in length from 24 min to 89 min. The transcripts of these interviews were analyzed using flexible inductive coding methods to identify the program's overall impacts, following the methods outlined by Deterding and Waters (58). Ethics exemption was granted by the University of Saskatchewan Human Research Ethics Board, given the evaluative focus of the project. The incorporation of animals in a therapeutic capacity was approved by the University of Saskatchewan Animal Research Ethics Board.

Three main themes emerged from the analysis, which were described as prison-specific therapy dog-facilitated experiences of comfort, support, and love. This was not surprising, given that these are common therapy dog program outcomes outside of a prison context. Unique here is that these are staff-identified insights, whereas the majority of the extant literature focuses on participant experiences. Thus, focusing our perspective article on how these three concepts are perceived uniquely within a prison-specific context by staff can be a potentially important contribution to forensic psychiatry and allied professions.

### 3.1. Comfort

According to the staff, the program has a temporary impact on the prison environment for the participants. They explained that the prison environment is typically characterized by hypervigilance, fear, and projected strength as participants feel the need to hide any signs of vulnerability to stay safe and maintain status. However, the presence of the program dogs helped ease these tensions and alter the prison environment. Staff observed that participants were able to “let their guard down” and “show vulnerability” without fear of judgment. One staff member shared the following:

Staff 2: So this morning when we were sitting outside, Participant 1 and Participant 2 were sitting chatting and Participant 1 said, and it was just something that came out, and he said “I don't even feel like I am in prison right now…I feel like I am cured.”

Another staff member similarly shared the following:

Staff 1: Same with Participant 5. He's like, he's got gang affiliations, he's quite high ranking in his gang, so for him to let that down [his guard] a little bit, is big.

Additionally, the staff observed that the sense of comfort facilitated by the non-judgmental comforting presence of the dogs allowed participants to experience and express emotions that they would not normally, and for some, had not done in a very long time.

Staff 3: So that's some empathy, which again would be huge for Participant 4. Cause that's a big issue for him. Cause he can't show it, you know, you get so used to being that, you know look at me, the big tough guy… he probably, probably a place he hasn't been since I would say he was a child.

#### 3.1.1. Comfort—insight for forensic psychiatry

Participating in the program offered participants a sense of comfort to explore their emotions and feelings with reduced fear of judgment. The culture and norms within the prison, alternatively, can create a disconnection between a participant and their emotions. This is especially important to substance-using individuals as disconnection from emotions and difficulty regulating them can lead to increased substance use (59, 60). In order to reduce the harm surrounding prisonization and how it can impact substance use, principles of compassionate care can be learned from the therapy dogs in this CAI. Recognizing complex emotions with compassion is a key element of compassionate care while reducing stigma reflects key principles of harm reduction as well (54, 61).

In forensic psychiatry, comfort is a key element to build rapport and trust with patients and is crucial for effective treatment (62–64). Animal-assisted interventions, with their attention to establishing participant comfort, have been found to be an effective and efficient way to achieve a therapeutic working alliance (23, 65, 66). Promoting comfort is also an anxiety management technique applied by forensic psychiatrists as is the promotion of relaxation, empathy, and fearlessness (67).

Our team has developed a Top 10 Resource for service providers, including in forensic psychiatry, to access evidence-informed outcomes that recognize the beneficial role of animals in the lives of individuals facing substance use concerns. Recognition of the positive benefits of human–animal interaction by service providers is a key, although under-recognized, way to build comfort with program clients. The resource is available at https://colleendell.com/top10.

### 3.2. Support

The staff members observed that having PAWSitive Support therapy dogs in the prison contributed to creating a supportive environment, which resulted in stronger relationships between the participants and prison staff. One staff shared (Staff 1) “More than anything probably [the program] is what motivated him but like, I think this helped him, like I said with his anxiety and with probably staying sober… and definitely helped with his relationship with staff.”

The staff members noted that the presence of therapy dogs during the program increased the participants' recognition of being supported within the institution, which in turn facilitated the development of trusting relationships between the participants and the staff.

Staff 1: He seemed to have more trust with me. Like cause when he came to me about getting out of the gang, it was like he came to the door… then he closed the door and “what do you think about maybe a transfer, so I can maybe distance myself, you know?”

Staff also thought that the program participants' trust with them was strengthened when they saw that prison staff supported the program, such as when one of the therapy dogs was permitted to visit a participant in the segregation unit.

Facilitator 1: Instead of fighting the institution… [the participants began] seeing it as something that was supporting them, and even… taking [therapy dog] to see [Participant 4] in] Seg yesterday,

Staff 1: You took [therapy dog]? Oh my gosh, that must have been so big for [Participant 4] …. I can't believe you got in there, that is amazing.

#### 3.2.1. Support—insight for forensic psychiatry

The therapy dogs provided a positive and supportive alternative to the traditional prison environment. This had a positive impact on the participants' relationships with the institutional staff. Research has shown that supportive relationships and strong therapeutic alliances can reduce the risk of substance use for patients in recovery (68, 69). Supportive relationships that advocate for the rights and dignity of clients also strongly align with compassionate care and harm reduction (52, 70). While there is little research on the concept of support within forensic psychiatry, some studies have shown that staff taking an interest in patients' progress can lead to lower levels of distress (71, 72). Additionally, the recovery literature emphasizes the importance of a supportive environment for exploring client therapeutic change. There is quite a bit of literature on the importance of the therapeutic alliance in forensic psychiatry, where it has been found to be a key element in developing a patient-centered practice and achieving better outcomes for patients (73–75).

Our team has created a resource in collaboration with the Canadian Centre on Substance Use and Addiction to help professionals incorporate animals into their practice to create a more supportive and client-centered environment. This resource includes common definitions in the field, a video series, and a resource guide on how to become animal-informed as a practitioner. This resource also incorporates the voices of people with lived experience of substance use whose animals supported them in their recovery, reflecting the harm reduction tenant of respecting the expertise of people with lived and living experience (55). You can access this resource at https://colleendell.com/pawsitivecanine.

### 3.3. Love

The staff reflected that participants in the PAWSitive Support program expressed gratitude for being a part of it and had positive feelings toward the prison for allowing them to take part in it. They felt that the institution cared for them and that they mattered. This is not a common sentiment within the broader culture of prisons.

Facilitator 1: One thing that came out really strong from everybody… was that they saw [Correctional Service Canada] as supporting and caring about them. Which was surprising to me, is not how I was really thinking about it but to allow the program into here, and to allow them to participate in it, they saw as, maybe CSC really does care.

Additionally, the staff observed that the program participants' interactions with the therapy dogs were non-judgmental, trusting, and loving. It provided them with an opportunity to see themselves beyond the externally and internally imposed labels of “criminal,” “lifer,” or “gang member.”

Staff 4: [Participant 4] was very surprised, somebody would do that for him, because he's a bad guy, he's in prison, “well those dogs don't know that I've done bad things, and they still love me,” they're jumping on them, they're licking them, they're hugging them, and to have that love with no strings attached, because they can't do anything for you, they can't do anything for the dogs, and they know that, but still the dogs love them. And you guys took time out of your world and your life, but there's a lot of other things you could be doing, and they see that too, like “wow, they actually came in here, and wanna spend this time with us, wow, maybe I'm not so bad. Maybe I am okay.”

#### 3.3.1. Love—insight for forensic psychiatry

Evidence-based literature tends to avoid discussing the concept of love. However, within the context of the PAWSitive Support program, institutional staff identified love as the participants' feeling of being cared for and valued. The concept of “mattering” is explored more commonly in literature than love. Research indicates that not feeling like one matters can contribute to increased criminal activity, mental health issues such as depression and suicidal tendencies, and a greater likelihood of substance use (76–79). Not feeling loved for and experiencing isolation within a prison environment can exacerbate these feelings (13, 80, 81). Harm reduction and compassionate care both reflect a need to address these experiences of isolation and support individuals without judgment (53, 61).

Our team is active on social media platforms (@PAWSitiveCanineConnections) and the Internet (www.colleendell.com), where access to our front-line experiences, research outcomes, and participant testimonials are regularly shared. This includes access to the Animal Memories magazine our office co-led in the development of, and which the Commissioner of Correctional Service Canada identified as “a remarkable collection of inmate stories about the importance of animals in their lives” (38). You can access the magazine at https://www.flipsnack.com/harperg/animal-memories.html.

## 4. Concluding remarks on therapy dogs, compassionate care, and harm reduction

Offering compassionate care in a prison setting is challenging, and establishing trust between participants and staff and within an institutional environment can seem impossible. One possible way to enhance the quality of care in forensic psychiatry is to take into account the insights of the Drumheller Institution staff and thoughts from PAWSitive Support facilitators who work with participants in this prison canine-assisted intervention program. According to the staff, the program's therapy dogs create a distinct and integrated sense of comfort, support, and love for participants—all elements of compassionate care. This, in turn, helps to establish some trust between participants and staff as well as the institution. Improving staff-participant relations is beneficial to creating a therapeutic alliance that supports participants' overall wellbeing and addresses the negative impacts of prisonization, such as substance use. By integrating this knowledge about human–animal interaction and insights into compassionate care into its practice, forensic psychiatry may be able to create a more positive and supportive environment for its patients, leading to better therapeutic outcomes, harm reduction for prisonization, and an improved standard of care.

It must also be noted that ensuring the welfare of the participating program dogs is crucial to a program's success. This is central to the offering of the PAWSitive Support program. Future research should consider collecting data over a longer term and include pre- and post-intervention measures, including mental health and substance use-specific outcome indicators. At the same time, moderating factors on outcomes need to be fully accounted for, such as participants' mental health symptoms and length of incarceration.

## Data availability statement

The original contributions presented in the study are included in the article/supplementary material, further inquiries can be directed to the corresponding author.

## Ethics statement

The perspective study involving human participants was reviewed and given ethics exemption by the University of Saskatchewan Human Research Ethics Board given its evaluation focus. The patients/participants provided their written informed consent to participate in this study. The animal study was reviewed and approved by University of Saskatchewan Animal Research Ethics Board. Written informed consent was obtained from the owners for the participation of their animals in this study.

## Author contributions

MG wrote the original draft of this manuscript. MG and CD contributed to the conceptualization and editing of the manuscript. CD, DC, GR, and MM all contributed sections of the manuscript. All authors contributed to manuscript revisions and approved the submitted version.
